# Comparison Among Endoscopic, Laparoscopic, and Open Resection for Relatively Small Gastric Gastrointestinal Stromal Tumors (<5 cm): A Bayesian Network Meta-Analysis

**DOI:** 10.3389/fonc.2021.672364

**Published:** 2021-11-29

**Authors:** Zhen Liu, Ziyang Zeng, Siwen Ouyang, Zimu Zhang, Juan Sun, Xianze Wang, Xin Ye, Weiming Kang, Jianchun Yu

**Affiliations:** Department of General Surgery, Peking Union Medical College Hospital, Chinese Academy of Medical Sciences and Peking Union Medical College, Beijing, China

**Keywords:** endoscopic resection, laparoscopic resection, open resection, gastric gastrointestinal stromal tumor, network meta-analysis

## Abstract

**Background:**

Endoscopic resection (ESR) is a novel minimally invasive procedure for superficial tumors. Its safety, efficiency, and outcome for gastric gastrointestinal stromal tumors (gGISTs) less than 5 cm remains unclear compared to laparoscopic resection (LAR) and open resection (ONR). The current network meta-analysis aimed to review and analyze the available evidence of this question.

**Methods:**

PubMed, Embase, Cochrane Library, and Web of Science databases were searched to identify eligible studies published up to July 6, 2020. The perioperative and long-term oncological outcomes among ESR, LAR, and ONR for gGIST (<5 cm) were estimated through the Bayesian network meta-analysis with a random-effect model.

**Results:**

Fifteen studies with 1,631 patients were included. ESR was associated with a shorter operative time [mean difference, MD: -36; 95% confidence interval, CI (-55, -16)], a higher rate of positive margin [odds ratio, OR: 5.1 × 10^10^, 95% CI (33, 2.5 × 10^32^)], and less costs [MD: -1 × 10^4^, 95% CI (-1.6 × 10^4^, -4.4 × 10^3^)] but similar time to resume flatus [MD: 0.52, 95% CI (-0.16, 1.1)] and diet [MD: -3.5, 95% CI (-5.6, -1.6)] compared to LAR. A higher rate of total complications [OR: 11, 95% CI (1.2, 140)] was observed in patients who received ESR compared to patients who received LAR. After excluding perforation from the total complication category, the difference of complication between ESR and LAR disappeared [OR: 0.87, 95% CI (0.22, 2.3)]. The recurrence rate [OR: 1.3, 95% CI (0.40, 4.5)] and disease-free survival [hazard ratio: 1.26, 95% CI (0.60, 2.63)] showed no significant difference between ESR and LAR. ESR was associated with better or equivalent perioperative and long-term outcomes compared to ONR, except for positive margin. A subgroup analysis (<2 and 2–5 cm) showed no significantly different results among these three procedures either.

**Conclusion:**

ESR was shown to be a safe and efficient alternative procedure to both LAR and ONR for gGISTs less than 2 cm and within 2–5 cm, respectively, without worsening the oncologic outcomes. However, preoperative assessment of tumor site is of importance for the determination of procedures regarding the increased incidence of a positive margin related to ESR.

## Introduction

Gastrointestinal stromal tumor (GIST), one of the most common mesenchymal tumors arising from the digestive tract, has the highest incidence in stomach (1). Until now, surgery is still the first option among treatments for primary gastric GIST (gGIST). Local resection with a clear margin and avoidance of tumor rupture could achieve a satisfying oncologic outcome for this kind of tumor due to its rare invasion of the lymph node or adjacent organ (2, 3), which provides the possibility for a minimally invasive resection.

Laparoscopic resection (LAR) has been recommended for selected gGISTs in favorable anatomical sites by the National Comprehensive Cancer Network guidelines (4) and additionally for tumors less than 5 cm by the Chinese Consensus Guidelines (5). Some studies reported that LAR was even safe and feasible for gGIST larger than 5 cm compared with open resection (ONR). Recently, endoscopic resection (ESR), with the superiority of maintaining the intact structure of the stomach, has also been demonstrated safe and effective for gGIST not larger than 5 cm when performed by an experienced endoscopists (6), but it is challenging for ESR to ensure R0 resection, and its specific complications, such as perforation and bleeding, may result in conversion to surgery (7). Up to date, a strong evidence-based impact of ESR on gGIST less than 5 cm is lacking.

In the current study, a Bayesian network meta-analysis was conducted to compare the perioperative and long-term oncological outcomes of ESR, LAR, and ONR for gGIST less than 5 cm.

## Materials and Methods

### Search Strategy and Study Selection

Two authors (ZL and ZZe) independently carried out a comprehensive systematic search on PubMed, Embase, Web of Science, and Cochrane Library using the following keywords: (“gastrointestinal stromal tumor”) and (“gastric” or “stomach”) and (“endoscop*” or “laparoscop*” or “open resection”). The searches were limited to articles that were published up to July 6, 2020.

The results were screened and identified by two authors (ZL and SO) according to the following criteria: (1) studies that compared any two or three of ESR, LAR, and ONR for patients with gGIST, (2) studies that included patients whose tumor diameter was less than 5 cm, (3) studies that included arms that had more than 10 cases of patients, (4) studies that provided perioperative outcomes and/or long-term survival outcomes (or sufficient information to estimate the corresponding parameters), and (5) when duplicate studies based on similar populations were identified, only the newest or largest study was included.

### Data Extraction and Quality Assessment

Two observers (ZL and JS) independently extracted data including the name of the first author, year of publication, period of study, country, sample size, age, sex, tumor size, operative time, intraoperative blood loss, positive margin, conversion, postoperative complication, first time to flatus, first time to diet, hospitalization, follow-up, recurrence, recurrence-related death, and disease-free survival of patients with gastric gastrointestinal stromal tumors. If the hazard ratio (HR) and 95% confidence interval (CI) were not provided in the included studies, we calculated these data from available data or from Kaplan–Meier survival curves using the methods reported by Tierney et al. (8). A third observer (ZZh) engaged in a discussion to resolve any controversial issues.

Two authors (ZZe and XW) independently assessed the quality of all the included studies using the Newcastle–Ottawa Quality Assessment Scale (NOS), and any discrepancies in the score were resolved by a discussion. The maximum score is nine points, and an article with a score equal to or more than six points was considered of high quality.

### Statistical Analysis

The mean difference (MD), odds ratio (OR), and HR with 95% credible interval (CrI, for network meta-analysis) or 95% CI (for traditional pairwise meta-analysis) were used to analyze continuous, dichotomous, and survival parameters, respectively. The HR and its corresponding 95% CI from Kaplan–Meier curves were extracted using Engauge Digitizer (version 4.1).

A Bayesian network meta-analysis with a random-effect model was performed using R software (3.6.1) with the GeMTC package (0.8-7) and rjags package (version 4-10) (9). The trace plot, density plot, and Brooks–Gelman–Rubin plot were employed to evaluate the convergency of the network process. The consistency model was recommended to conduct a further analysis due to the absence of a significant difference when compared to the inconsistency model. The node-split method assessing the direct and indirect pairs also suggested the absence of inconsistency. The parameter *I* ^2^ was used to assess the heterogeneity between studies. The estimated relative effects were represented in a forest plot, and ranking plots were drawn based on the distribution of the ranking probability of each procedure. A procedure with a higher probability is preferred to be recommended. The potential publication bias was assessed by comparison-adjusted funnel plot using Stata 14.0 (Stata Corporation, Texas, USA).

The rate of positive margin and conversion between ESR and LAR were compared directly using R software (3.6.1) with the meta package (4.13-0) (10). The data of ONR was not available for a subgroup analysis of tumors within 2–5 cm, the effects of which were also estimated by a direct meta-analysis.

## Results

### Study Selection and Network Assumptions

**Figure 1:**
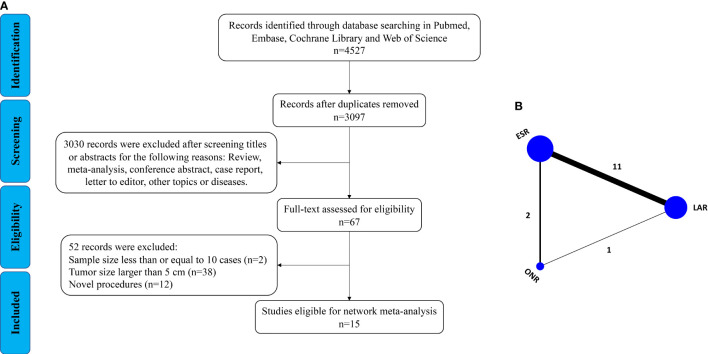
Flow chart of the **(A)** search strategy and **(B)** study design.

As shown in **Figure 1A**, a total of 4,527 articles were retrieved by the initial search strategy. After checking for duplicates and screening the irrelevant topics through the titles and abstracts, 4,460 pieces of the records were removed. Then, 52 studies were excluded after the full-text assessment. Finally, 15 studies (11–25) of 1,631 patients were included in this network meta-analysis. The characteristics of the eligible studies are summarized in **Table 1**. There were 555 patients who received LAR, 911 patients who received ESR, and 165 patients who received ONR. The NOS score of the studies ranged from 6 to 8, indicating the relatively high quality of the methodology.

**Table 1:** Summary of eligible studies.

Study	Procedure	Sample size/sex (male)	Age (mean ± SD)	Tumor size (mean ± SD)	Positive margin	Conversion^a^	Complication (a/b/c)^b^	Oncological events (a/b/c)^c^	Disease-free survival	Follow-up	NOS
Zhao2019	ESR	85/31	57.01 ± 9.66	1.6 ± 0.88	4	0	5/5/85	0/0/85	NA	NA	6
Zhao2019	LAR	64/29	57.77 ± 10.36	3.13 ± 1.11	0	1^d^	5/5/64	0/0/64	NA		
Zhao2019	ONR	67/36	60.6 ± 10.9	3.27 ± 1.27	0	0	11/11/67	0/0/67	NA		
Dong2019	ESR	45/24	56.3 ± 9.8	2.6 ± 0.7	1	1^e^	16/0/45	2/0/45	Reference	75.5 (6–108) mo	8
Dong2019	LAR	45/26	55.8 ± 9.9	2.9 ± 0.8	0	0	0/0/45	1/0/45	2.29 (0.36, 14.43)	65 (6–124) mo	
Yin2018	ESR	46/23	60.09 ± 10.95	2.04 ± 0.87	1	0	7/4/46	0/0/46	NA	69.5 (12–100) mo	6
Yin2018	LAR	30/12	54.47 ± 10.78	3.7 ± 1.16	0	0	3/3/30	1/0/30	NA		
Chen2018	ESR	35/12	56.46 ± 11.17	2.67 ± 0.62	0	NA	1/1/35	2/0/35	Reference	45 (17–60) mo	8
Chen2018	LAR	66/27	60.41 ± 9.8	3.06 ± 0.6	0	NA	4/4/66	6/2/66	1.15 (0.65, 1.97)	60 (15–60) mo	
Zuo2017	ESR	41/18	52.2 ± 4.1	NA	0	1^f^	NA	NA	NA	0.5–4 y	6
Zuo2017	ONR	36/21	61.1 ± 6.7	NA	0	0	NA	NA	NA		
Meng2017	ESR	75/35	50.64 ± 11.22	1.44 ± 0.65	NA	NA	2/NA/75	2/0/75	Reference	3.41 ± 1.37 y	8
Meng2017	LAR	51/25	54.53 ± 11.06	1.46 ± 0.62	NA	NA	1/NA/51	1/0/51	1.67 (0.69, 3.8)		
Dai2017	ESR	262/106	57 ± 10.32	1.33 ± 0.78	NA	2^g^	15/12/260	2/NA/219	NA	32.99 ± 14.39 mo	7
Dai2017	LAR	73/30	57.95 ± 11.89	1.97 ± 0.93	NA	1^h^	2/2/73	0/NA/62	NA	35.32 ± 13.28 mo	
Balde2017	ESR	30/14	49.9 ± 11.9	1.54 ± 0.39	3	NA	8/6/30	2/0/30	Reference	57.9 ± 28.9 mo	8
Balde2017	LAR	30/14	48 ± 13.2	1.46 ± 0.7	0	NA	1/1/30	0/0/30	7.51 (0.62, 91.46)		
Wang2016	ESR	35/25	55 ± 14	1.3 ± 0.5	NA	0	35/0/35	0/0/35	NA	1–72 mo	7
Wang2016	LAR	33/20	56 ± 14	1.6 ± 0.4	NA	0	4/4/33	0/0/33	NA		
Meng2016	ESR	27/11	49.15 ± 10.31	1.18 ± 0.27	NA	NA	5/NA/27	1/0/11	Reference	7 (3–24) mo	7
Meng2016	LAR	48/19	53.17 ± 12.04	1.2 ± 0.22	NA	NA	2/NA/48	2/0/17	0.2 (0.05, 1.42)	6 (3–59) mo	
Wu2016	ESR	50/28	NA	NA	0	NA	50/0/50	0/0/50	NA	1 mo	8
Wu2016	LAR	42/23	NA	NA	3	NA	2/2/42	0/0/42	NA		
Huang2014	ESR	32/NA	NA	NA	0	0	0/0/32	0/0/32	NA	1 mo	6
Huang2014	LAR	30/NA	NA	NA	0	2^i^	1/1/30	0/0/30	NA		
Wang2011	ESR	66/31	44.64 ± 10.76	1.32 ± 0.68	NA	1^j^	32/17/66	NA	NA	NA	7
Wang2011	LAR	43/23	41.35 ± 9.97	1.17 ± 0.77	NA	0	6/6/43	NA	NA		
Feng2015	ESR	50/24	NA	NA	0	0	20/NA/50	0/0/50	NA	32 (12–65) mo	6
Feng2015	ONR	40/25	NA	NA	0	0	2/NA/40	0/0/40	NA		
Shen2015	ESR	32/15	60.54 ± 10.64	1.7 ± 0.36	0	1^k^	6/5/32	1/0/32	NA	31.5 (2–53) mo	7
Shen2015	ONR	22/11	55 ± 9.43	1.82 ± 0.2	0	0	3/3/22	1/0/22	NA	38.5 (5–50) mo	

ESR, endoscopic resection; LAR, laparoscopic resection; ONR, open resection; NA, not available; y, year; mo, month; NOS, Newcastle–Ottawa Quality Assessment Scale.

Positive margin was defined as a microscopically positive resection margin or visually positive resection margin.

a Reason of conversion to other methods.

b Complications: a, total complications; b, complications excluding perforation; c, sample size.

c Oncological events: a, recurrence; b, recurrence-related death; c, sample size.

d Not available.

e One patient with a 4-cm tumor at the antrum and who received endoscopic resection was transferred to laparoscopic resection due to incomplete resection of the large tumor.

f One patient who received endoscopic resection was transferred to open resection due to severe intraoperative bleeding.

g Two patients who received endoscopic resection were finally transferred to laparoscopic resection due to the close adhesion of tumors to the gastric wall.

h One woman with a 3.5-cm tumor in the cardia and who received laparoscopic resection was finally transferred to open resection due to a positive margin.

i Two patients who received laparoscopic resection were transferred half-way to open resection due to the unfavorable sites of the tumors located in the posterior wall of the fundus near the cardia.

j One patient who received endoscopic resection was transferred to open resection due to the unfavorable site of the tumor located in the fundus near the dome of the stomach.

k One case of a patient who experienced perforation caused by endoscopic resection was converted to laparoscopic repair of the stomach wall.

Then, a Bayesian approach with random-effect model was employed to conduct the network analysis (**Figure 1B**). The trace plots, density plots, and Brooks–Gelman–Rubin plots showed a good convergence of the process (**Supplementary Figure 1**). According to the node-splitting analysis, no significant inconsistency was detected between direct and indirect comparisons (*P* > 0.05). The network results were shown as follows:

### Operative Time

**Figure 2:**
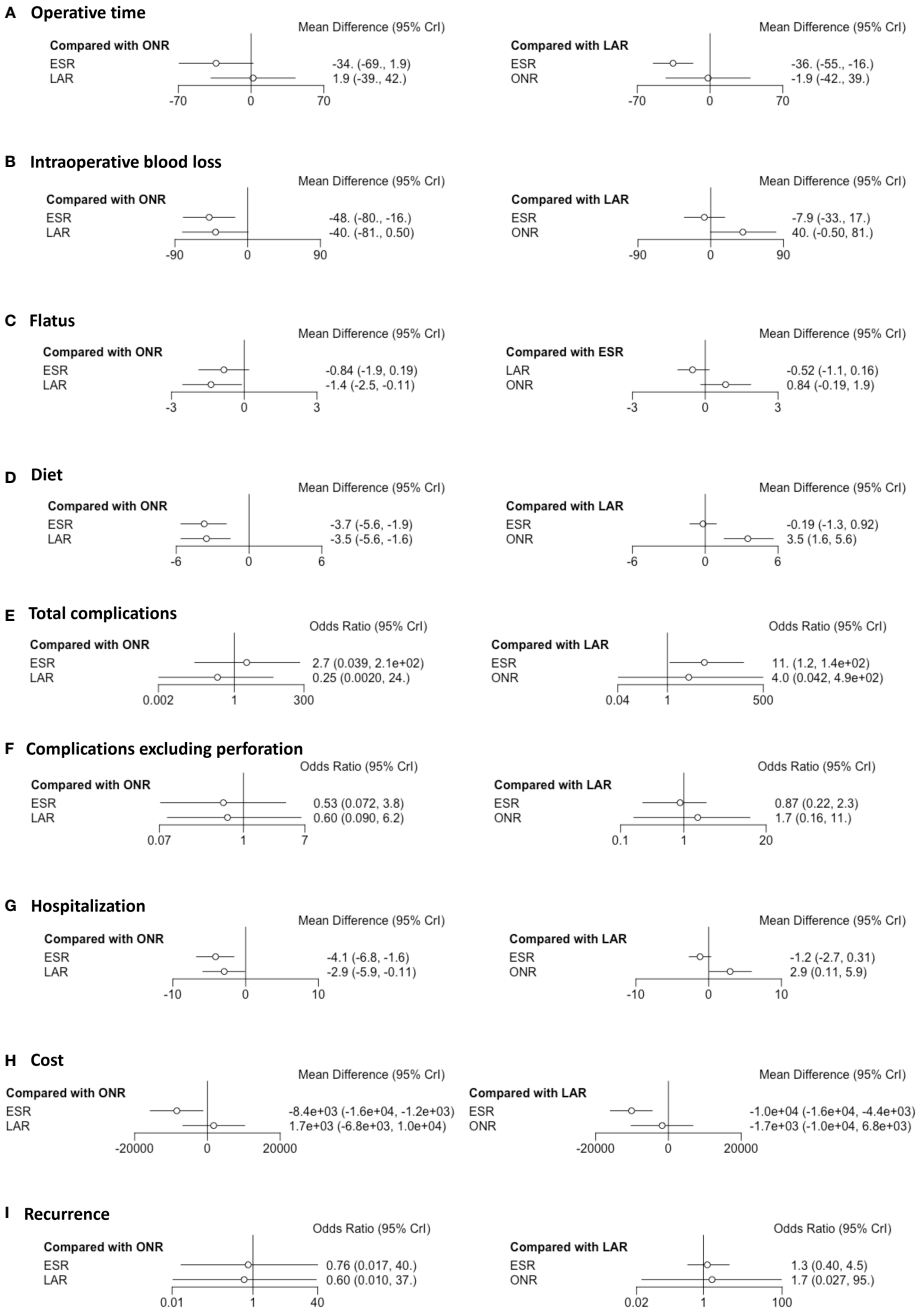
Forest plots of the network meta-analysis between endoscopic resection, laparoscopic resection, and open resection. The **(A)** operative time, **(B)** intraoperative blood loss, **(C)** flatus, **(D)** diet, **(E)** total complications, **(F)** complications excluding perforation, **(G)** hospitalization, **(H)** cost and **(I)** recurrence were analyzed respectively.

**Figure 3:**
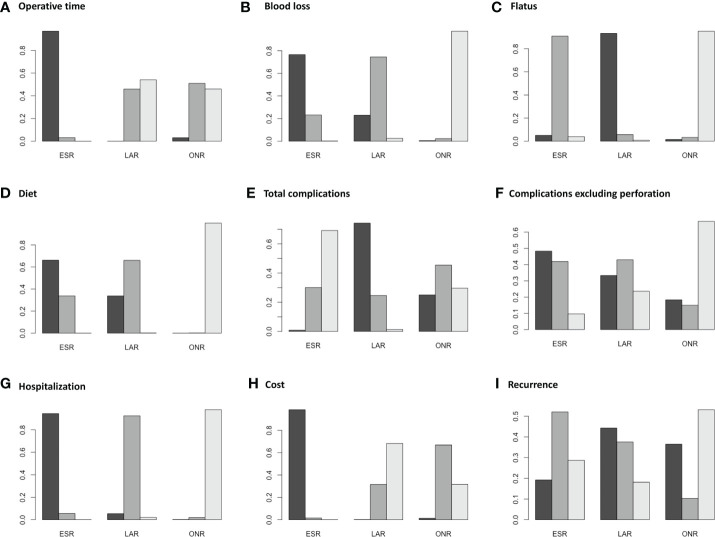
Ranking plots for the pooled data of **(A)** operative time, **(B)** intraoperative blood loss, **(C)** flatus, **(D)** diet, **(E)** total complications, **(F)** complications excluding perforation, **(G)** hospitalization, **(H)** cost and **(I)** recurrence between endoscopic resection, laparoscopic resection, and open resection.

Fourteen studies were available for operative time. As shown in **Figure 2A**, the operative time of ESR was shorter than that of LAR [MD: -36, 95% CI (-55, -16)] but not significantly different from that of ONR [MD: -34, 95% CI (-69, 1.9)]. The difference between LAR and ONR was not statistically significant [MD: 1.9, 95% CI (-39, 42)]. The ranking plot (**Figure 3A**) represented ESR as the first choice in regard to operative time because of its highest probability of being ranked first, followed by the juxtaposition of ONR and LAR.

### Intraoperative Blood Loss

Eight studies were available. ESR was associated with less intraoperative blood loss compared to ONR [MD: -48, 95% CI (-80, -16)] (**Figure 2B**). The difference between ESR and LAR [MD: -7.9, 95% CI (-33, 17)] was not statistically significant, and neither that between LAR and ONR [MD: -40, 95% CI (-81, 0.5)]. According to the ranking plot, ESR was most likely to be the first choice in regard to intraoperative blood loss, followed by LAR and ONR (**Figure 3B**).

### Positive Margin and Tumor Rupture

**Figure 4:**
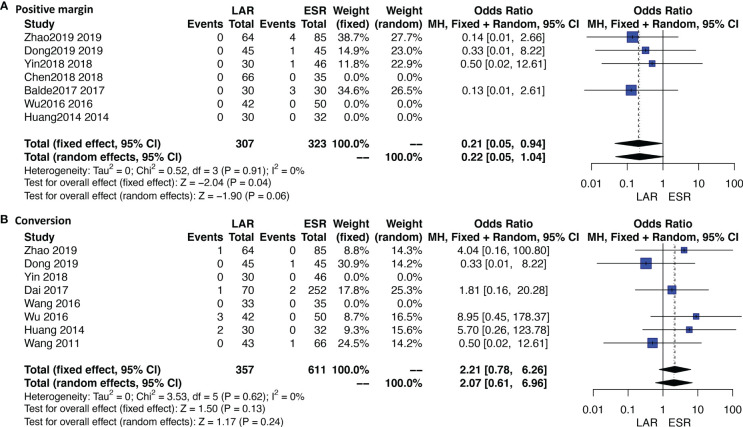
Forest plots of direct meta-analysis comparing rates of **(A)** positive margin and **(B)** conversion between endoscopic resection and laparoscopic resection.

Ten studies were available. No patient who received ONR or LAR experienced a positive margin or tumor rupture. ESR showed a significantly higher rate of a positive margin than LAR [OR: 0.21, 95% CI (0.05, 0.94); *I* ^2^ = 0%, *P* = 0.91, fixed-effect model] (**Figure 4A**). Data of rupture were available in four studies, and rupture occurred in only one patient who received ESR.

### Conversion Rate

Eleven studies were available. A total of 10 patients who received ESR were transferred to other methods. The detailed reasons of conversion are listed under **Table 1**. Four out of six patients were transferred to LAR or ONR due to the unfavorable sites of tumors; the other two reasons were severe bleeding and perforation. Three out of four patients who received LAR were transferred to ONR due to the unfavorable sites; the other one is not available. The conversion rate was not significantly different between ESR and LAR [OR: 2.21, 95% CI (0.78, 6.26); *I* ^2^ = 0%, *P* = 0.62, fixed-effect model] (**Figure 4B**).

### Time to Resume Flatus and Diet

Four studies were available for time to resume flatus. Patients who received ESR did not resume to flatus earlier than patients who received ONR [MD: -0.84, 95% CI (-1.9, 0.19)] or LAR [MD: 0.52, 95% CI (-0.16, 1.1)]. LAR was associated with a shorter time to resume flatus when compared with ONR [MD: -1.4, 95% CI (-2.5, -0.11)] (**Figure 2C**). The ranking plot showed that LAR had the highest probability of being ranked as the first choice, followed by ESR and ONR (**Figure 3C**).

Seven studies were available for time to resume diet. Patients who received ESR [MD: -3.7, 95% CI (-5.6, -1.9)] and LAR [MD: -3.5, 95% CI (-5.6, -1.6)] resumed diet earlier than patients who received ONR (**Figure 2D**). There was no significant difference between ESR and LAR [MD: -0.19, 95% CI (-1.3, 0.92)]. The ranking plot recommended ESR as the first choice, followed by LAR and ONR (**Figure 3D**).

### Complications

Fourteen studies were available for total complications. The total complication rate of ESR was higher than that of LAR [OR: 11, 95% CI (1.2, 140)] (**Figure 2E**). No significant difference was found between ESR and ONR [OR: 2.7, 95% CI (0.039, 210)] nor between LAR and ONR [OR: 0.25, 95% CI (0.002, 24)]. The ranking plot recommended LAR as the first choice of procedure, followed by ONR and ESR (**Figure 3E**).

After excluding perforation from the complication category (11 studies available), the complication rate did not differ significantly among these three procedures [ESR *vs.* ONR: OR: 0.53, 95% CI (0.072, 3.8); LAR *vs.* ONR: OR: 0.60, 95% CI (0.09, 6.2); ESR *vs.* LAR: OR: 0.87, 95% CI (0.22, 2.3)] (**Figure 2F**). The ranking plot juxtaposed ESR as the first choice of procedure in regard to complications, followed by LAR and ONR (**Figure 3F**).

### Hospitalization and Cost

Fifteen studies were available for hospitalization. ESR [MD: -4.1, 95% CI (-6.8, -1.6)] and LAR [MD: -2.9, 95% CI (-5.9, -0.11)] were associated with shorter hospitalization when compared to ONR (**Figure 2G**), but the difference between ESR and LAR was not significant [MD: -1.2, 95% CI (-2.7, 0.31)]. The ranking plot recommended ESR as the first choice of procedure followed by LAR and ONR (**Figure 3G**).

Seven studies were available. ESR cost significantly less than ONR [MD: -8.4 × 10^-3^, 95% CI (-1.6 × 10^4^, -1.2 × 10^3^)] and LAR [MD: -1 × 10^4^, 95% CI (-1.6 × 10^4^, -4.4 × 10^3^)], while the difference was not significant between LAR and ONR [MD: 1.7 × 10^3^, 95% CI (-6.8 × 10^3^, 1 × 10^4^)] (**Figure 2H**). The ranking plot suggested ESR as the first choice of procedure, followed by ONR and LAR (**Figure 3H**).

### Recurrence Rate

Thirteen studies were available. The recurrence rate showed no significant difference among these three procedures [ESR *vs.* ONR: OR: 0.76, 95% CI (0.017, 40); LAR *vs.* ONR: OR: 0.60, 95% CI (0.010, 37); ESR *vs.* LAR: OR: 1.3, 95% CI (0.40, 4.5)] (**Figure 2I**). The ranking plot showed LAR as the first choice, followed by ESR and ONR (**Figure 3I**).

### Subgroup Analysis According to Tumor Size

**Figure 5:**
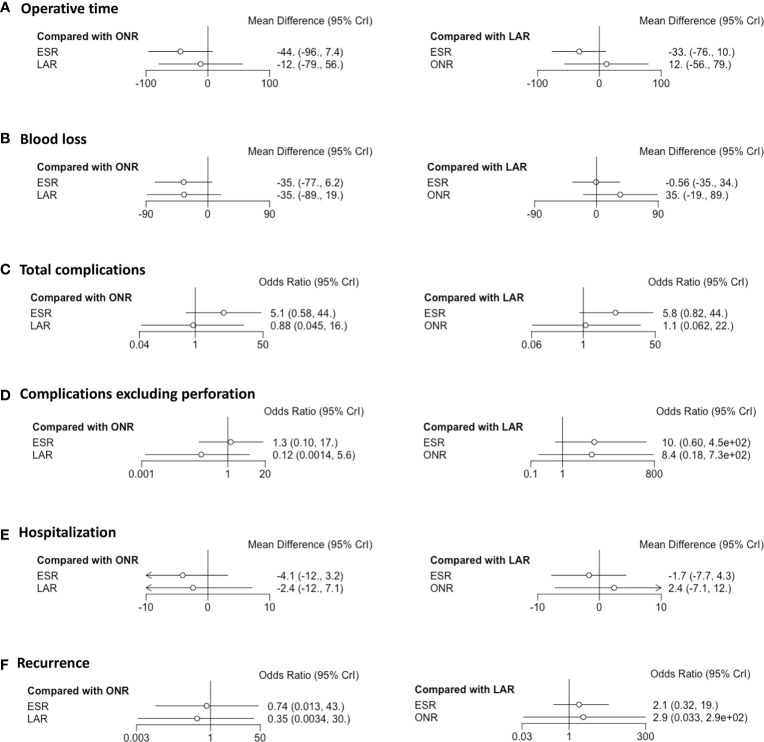
Forest plots illustrating the results of the subgroup analysis (tumor size < 2cm). The **(A)** operative time, **(B)** intraoperative blood loss, **(C)** total complications, **(D)** complications excluding perforation, **(E)** hospitalization and **(F)** recurrence were analyzed respectively.

**Figure 6:**
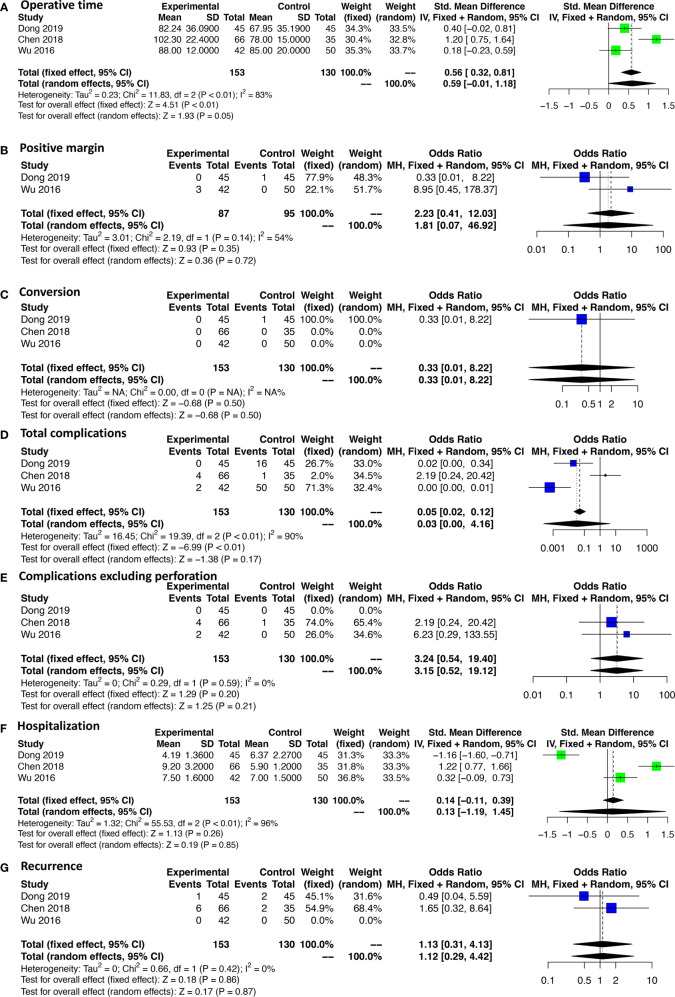
est plots illustrating the results of the subgroup analysis (tumor size 2-5cm). The **(A)** operative time, **(B)** positive margin rate, **(C)** conversion rate, **(D)** total complications, **(E)** complications excluding perforation, **(F)** hospitalization and **(G)** recurrence were analyzed respectively.

A subgroup analysis was performed according to the cutoff point of tumor size (<2 and 2–5 cm). ESR, LAR, and ONR showed no significant difference for tumors less than 2 cm in regard to operative time, intraoperative blood loss, rates of complications, and complications excluding perforation, hospitalization, and recurrence rate (**Figure 5** and **Supplementary Figure 2**). Data were only available for comparison of ESR and LAR for tumors between 2 and 5 cm, which also revealed a non-significant difference in regard to operative time, rate of positive margin and conversion, rate of total complications, and complications excluding perforation, hospitalization, and recurrence rate (**Figure 6**).

### Disease-Free Survival

**Figure 7:**
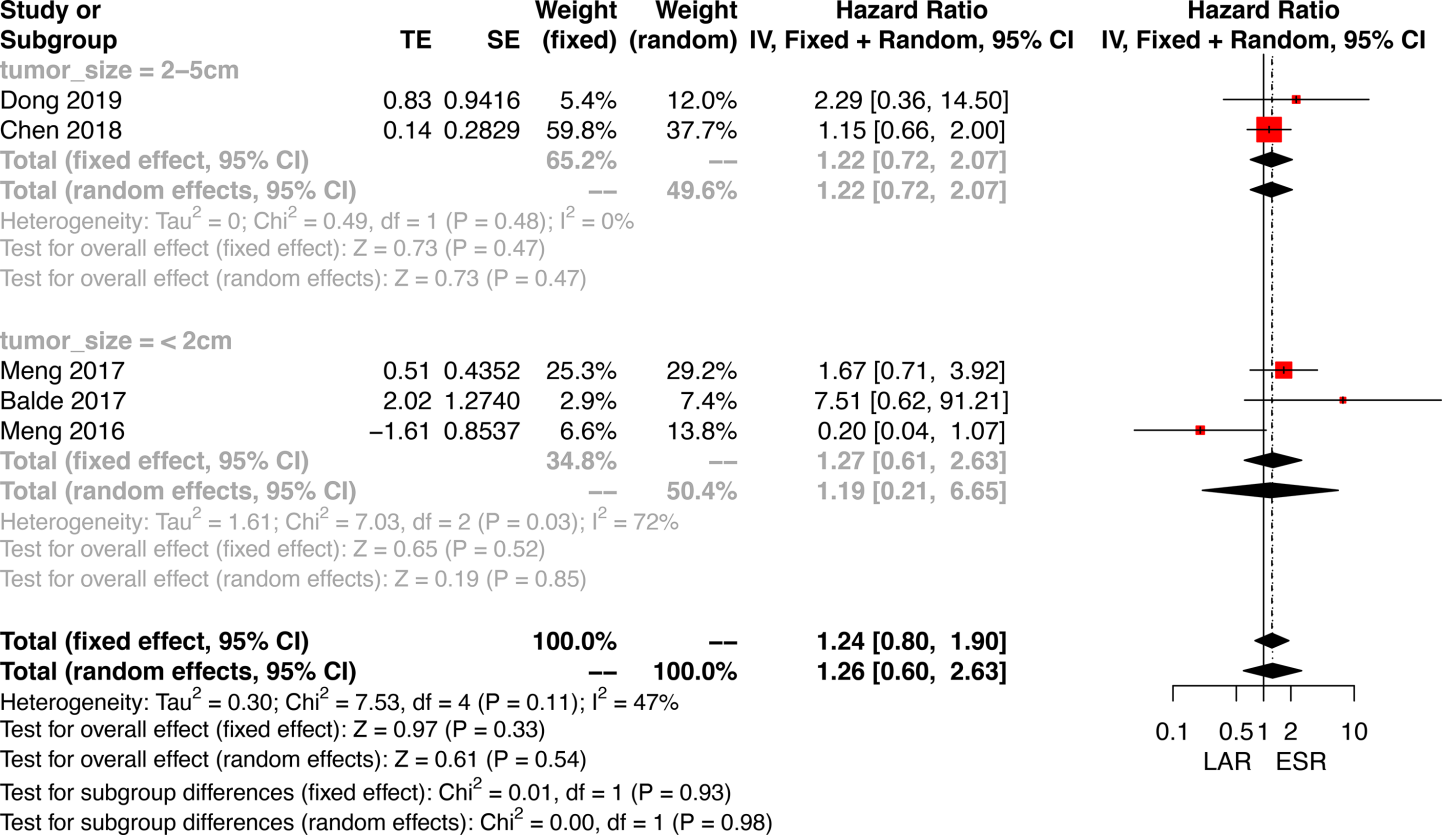
Forest plots illustrating the disease-free survival between endoscopic resection and laparoscopic resection.

Data on disease-free survival (DFS) was only available in five studies comparing ESR and LAR. Thus, a direct pair comparison (**Figure 7**) was conducted, and no significant difference of DFS was observed between ESR and LAR [HR: 1.26, 95% CI (0.60, 2.63)] according to the random-effect model. A subgroup analysis was performed in accordance of the cutoff point of tumor size (<2 and 2–5 cm). The DFS of ESR and LAR showed no significant difference in both subgroups [<2 cm: HR: 1.19, 95% CI (0.21, 6.65); 2–5 cm: HR: 1.22, 95% CI (0.72, 2.07)] (**Figure 7**).

### Publication Bias and Sensitivity Analysis

**Figure 8:**
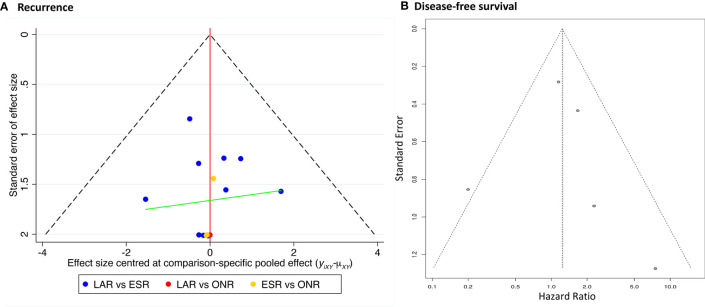
Publication bias of **(A)** recurrence rate and **(B)** disease-free survival.

The comparison-adjusted funnel plot was employed to assess the potential publication bias for recurrence rate and DFS (**Figure 8**). The funnel plots were visually symmetric, but small study effects might exist. Then, each study was eliminated sequentially, and the results of DFS between ESR and LAR did not change accordingly, which, in addition, confirmed the credibility of the current conclusion (**Supplementary Figure 3**).

## Discussion

The advances in minimally invasive surgery expanded LAR as a reliable alternative to traditional ONR for gGIST. The suitability of LAR for gGIST less than 5 cm has been reported by a series of studies (26–28). Recently, several studies have shown that ESR, a novel minimally invasive procedure, is safe and feasible for certain gGISTs (29, 30). A previous meta-analysis conducted by Wang et al. (31) has shown that there was no significant difference between ESR and LAR in terms of blood loss, hospitalization, time to flatus, time to liquid diet, and rate of postoperative complications for gGIST, but the survival has not been analyzed. On the contrary, in the meta-analysis of Zhu, ESR was associated with a shorter operative time, less intraoperative bleeding, earlier time to diet, shorter hospitalization, and less cost when compared to LAR (32). The unbalanced baseline between ESR and LAR has been considered as one of the sources of bias which might result in the contrary results detailed above. To verify this point of view, a recent case-matched study including 90 patients reported that, in contrast, LAR was better than ER for 2–5-cm gGISTs due to its lower complication rate and shorter hospitalization time (12). Thus, a stronger evidence-based network meta-analysis is needed to clarify this controversy. In the current study, 15 studies were included, and a subgroup analysis was conducted according to tumor size (<2 and 2–5 cm). ESR was demonstrated as safe and efficient as LAR and ONR without worsening the DFS for gGISTs either less than 2 cm or within 2–5 cm.

ESR is generally recommended for the advantage of causing less trauma because of its operation within the mucosa or submucosa and of reserving the integrity of the stomach, which improves the life quality of the patients (33). In the current study, ESR was associated with a shorter operative time but had a similar time to resume flatus and diet compared with LAR. It is reported that the high incidence of ESR-associated perforation of the gastric wall prolonged the postoperative hospitalization of patients (12, 18). Moreover, perforation and bleeding caused by ESR may also increase the risk of conversion to surgery assistance. The current results showed that a high rate of perforation was observed in patients who received ESR, which was associated with non-significant intraoperative blood loss, conversion rate, and hospitalization but less costs compared to LAR. A total of 10 patients experienced conversion to other methods. Two patients who received ESR were transferred to other methods due to perforation and bleeding, respectively, but the majority of reasons of conversion were unfavorable sites of tumors for both ESR and LAR, which indicate the necessity of a sufficient and precise assessment of the tumor site before performing ESR or LAR.

What is more, a higher rate of total complications was observed in patients who received ESR compared to LAR. Some included studies counted perforation into the complication category, but some authors holding the point of view that perforation can be successfully managed by titanium clips did not take perforation as a kind of complication, so complications were compared again after excluding perforation, which showed no significant difference between ESR and LAR. In addition, ESR and LAR both showed equal or better perioperative outcomes compared with ONR, except the rate of positive margin and conversion, which are primary considerations for the choice of procedures because of the concern for oncological safety.

Obtaining a completely negative margin and avoidance of tumor rupture are of great importance for surgical procedures (34). R1 margin was reported to be strongly related with tumor rupture (35). In the current study, a higher rate of R1 margin was observed in the ESR group compared to LAR. Data on rupture were available in four studies, and rupture occurred only in one patient who received ESR, so an analysis was not performed. Recently, an observational study (36) analyzing 908 GIST patients after surgery from a randomized phase III trial showed that, when including tumor rupture into its category, R1 was associated with worse overall survival of GISTs either with or without imatinib adjuvant therapy, but the difference in overall survival between R1 and R0 disappeared after excluding tumor rupture from R1 category. Similar results, that R1 did not impact survival, have been reported by other studies (37, 38).

Up to date, LAR has been demonstrated to have equivalent long-term oncological outcomes for gGIST compared to ONR (39–41). However, evidence of the long-term safety of ESR for gGIST is lacking yet. The current results showed that the recurrence rate and DFS were equal among ESR, LAR, and ONR despite the higher rate of R1 margin caused by ESR, which supported the previous conclusion that R1 margin did not impact the survival of GIST and demonstrated that ESR was suitable and safe for gGIST less than 5 cm in the premise of avoidance of tumor rupture. However, heterogeneity was observed in the comparison of recurrence rate and DFS, which we partly attributed to the discrepancy of the pathological features of gGISTs between studies as well as the unbalanced baseline between arms.

It is generally considered challenging to perform ESR for gGISTs with a larger size or arising from muscularis propria or unfavorable sites. Subgroup analysis, controlling confounding in some extent, should have been performed to clarify the impact of these factors, among which, however, only tumor size was available in the current study. Studies have been divided into two subgroups according to the cutoff point of tumor size (<2 and 2–5 cm). ESR, LAR, and ONR showed no significant difference in terms of operative time, intraoperative blood loss, rates of complications, and complications excluding perforation, hospitalization, recurrence rate, and DFS for tumors less than 2 cm. Three studies comparing ESR and LAR were available for tumors between 2 and 5 cm, one (Dong et al.) (12) of which was a case-matched study that was considered able to provide a more reliable result that LAR had a lower complication rate and shorter hospitalization time than ESR for gGISTs between 2 and 5 cm. However, the pooled results showed that operative time, rate of positive margin and conversion, rate of total complications, and complications excluding perforation, hospitalization, recurrence rate, and DFS had no significant difference between ESR and LAR for tumors between 2 and 5 cm. Thus, further well-designed studies focusing on the safety and efficiency of ESR for gGIST between 2 and 5 cm are needed.

Several limitations exist in this comprehensive network meta-analysis. First of all, bias of confounding and selection might exist because of the fact that all the included studies were retrospectively designed, through which randomization was absent, except for two studies that were designed by propensity score matching method—for example, tumors arising from different sites and layers of the stomach in each included study might lead to heterogeneity between studies. The diverse endoscopic approaches included in the ESR category, such as endoscopic submucosal dissection, endoscopic submucosal tunnel dissection, and endoscopic full-thickness resection that are practically performed for tumors arising from different layers of the stomach wall, might cause heterogeneity between studies. Second, the exact oncological outcomes of each patient with a positive margin were not reported in the included studies, although the total recurrence rate and DFS were not significantly different between procedures. Third, the observed heterogeneity in the current study was significantly reduced in certain subgroup analyses performed according to cutoff point of tumor size, but other unclarified confounding still existed. Fourth, the clinical heterogeneity caused by non-randomized allocation may lead to small study effects. Some included studies did not have a large sample size, although those with less than 10 cases had already been excluded. The selective patients in small studies might lead to clinical heterogeneity between small and large studies. The selective reporting of favorable outcomes in small studies might also lead to a publication bias. Fifth, all of those included studies were performed in China. Thus, large-sample-sized randomized controlled trials from multi- and transnational centers are needed to validate the current results.

## Conclusion

Endoscopic resection is shown to be a safe and efficient alternative procedure to both laparoscopic and open resection for gastric gastrointestinal stromal tumors less than 2 cm and within 2–5 cm, respectively, without worsening the oncological outcomes. Nevertheless, preoperative assessment of tumor site is of importance for the determination of procedures regarding the increased incidence of a positive margin and perforation related to ESR. Validation from future high-quality studies focusing on the impact of endoscopic resection for tumors within 2–5 cm is needed.

## Data Availability Statement

The original contributions presented in the study are included in the article/**Supplementary Material**. Further inquiries can be directed to the corresponding author.

## Author Contributions

WK, XY, and ZL contributed to the concept and design. ZL, ZZe, SO, and XW contributed to literature search and extracting of data. ZL, JS, and ZZh analyzed and interpreted the data. ZL drafted the manuscript. WK and JY critically revised and gave final approval of the manuscript. All authors contributed to the article and approved the submitted version.

## Funding

This study was supported in part by grants from 1. CSCO-ROCHE Research Fund (No. Y-2019 Roche-015); 2. Beijing Xisike Clinical Oncology Research Foundation (Y-HS2019-43); 3. Wu Jieping Medical Foundation (No. 320. 6750.19020); 4. CAMS Innovation Fund for Medical Sciences (2020-I2M-C&T-B-027).

## Conflict of Interest

The authors declare that this study received funding from CSCO-ROCHE. The funder was not involved in the study design, collection, analysis, interpretation of data, the writing of this article or the decision to submit it for publication.

The authors declare that the research was conducted in the absence of any commercial or financial relationships that could be construed as a potential conflict of interest.

## Publisher’s Note

All claims expressed in this article are solely those of the authors and do not necessarily represent those of their affiliated organizations, or those of the publisher, the editors and the reviewers. Any product that may be evaluated in this article, or claim that may be made by its manufacturer, is not guaranteed or endorsed by the publisher.

## Supplementary Material

Supplementary Figure 1 Plots of trace, density, and Brooks–Gelman–Rubin for the network meta-analysis.

Supplementary Figure 2 Plots of trace, density, and Brooks–Gelman–Rubin for the subgroup analysis (tumor size <2 cm).

Supplementary Figure 3 Sensitivity analysis.

## Abbreviations

GIST, gastrointestinal stromal tumor; ESR, endoscopic resection; LAR, laparoscopic resection; ONR, open resection; NCCN, National Comprehensive Cancer Network; NOS, Newcastle–Ottawa Quality Assessment Scale; HR, hazard ratio; OR, odds ratio; MD, mean difference; CI, confidence interval; CrI, credible interval; DFS, disease-free survival.
