# Supplementary figures and images for: Comparison Among Endoscopic, Laparoscopic, and Open Resection for Relatively Small Gastric Gastrointestinal Stromal Tumors (<5 cm): A Bayesian Network Meta-Analysis
Source: Front Oncol. 2021 Nov 29;11:672364. doi: 10.3389/fonc.2021.672364 (PMC8667731; doi:10.3389/fonc.2021.672364)

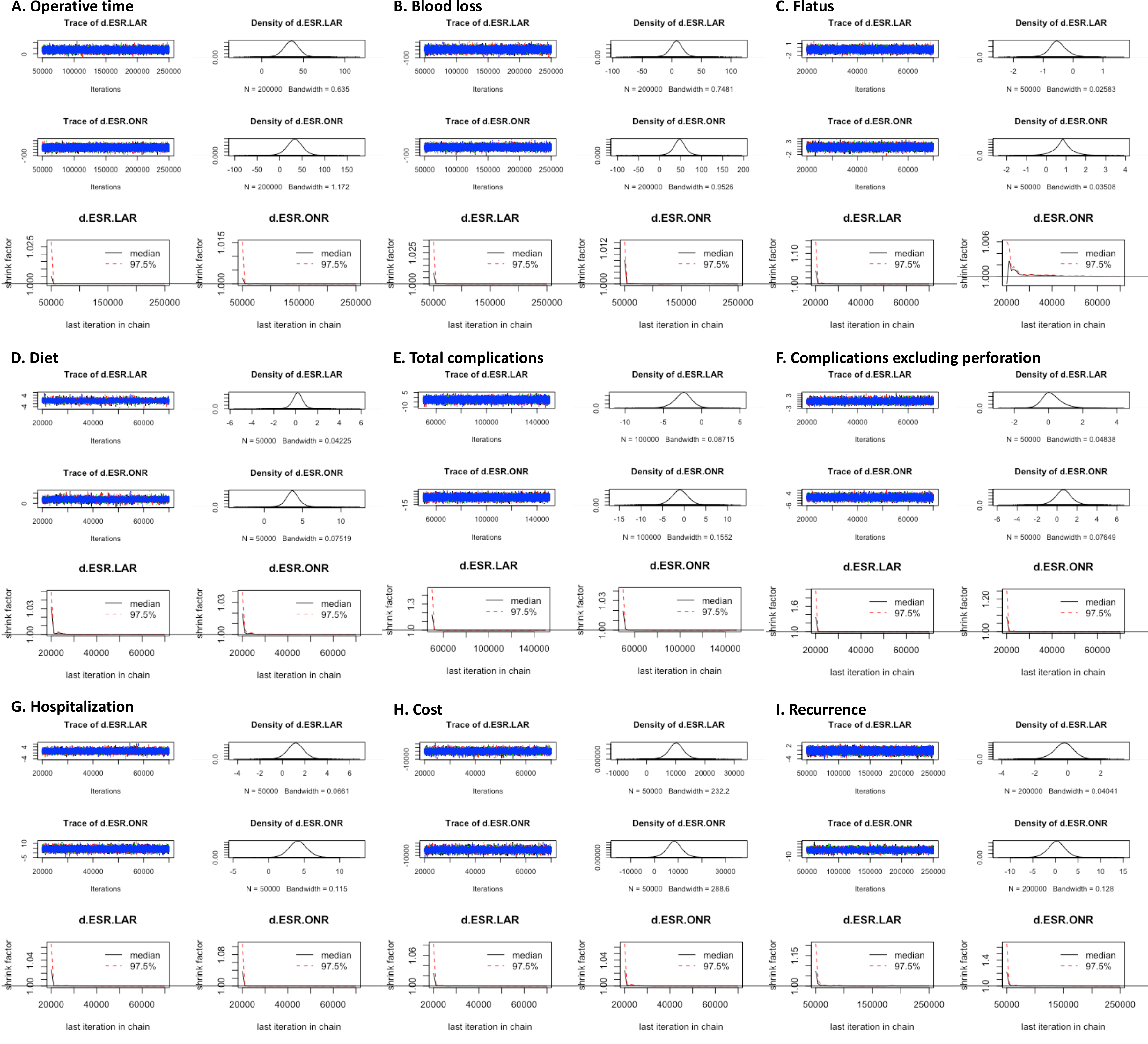

Supplement: Supplementary Figure 1 — Plots of trace, density, and Brooks–Gelman–Rubin for the network meta-analysis. [file Image_1.tif]

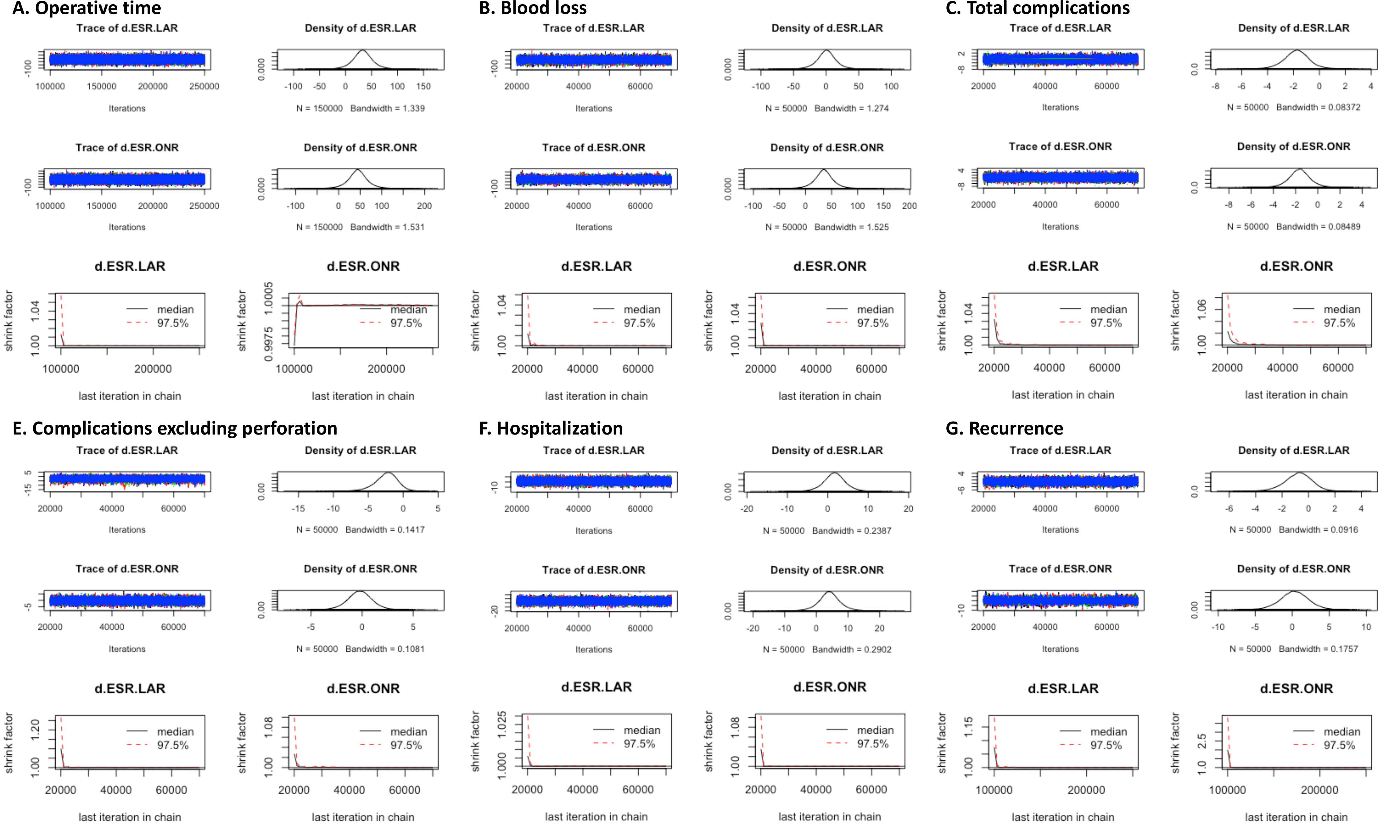

Supplement: Supplementary Figure 2 — Plots of trace, density, and Brooks–Gelman–Rubin for the subgroup analysis (tumor size <2 cm). [file Image_2.tif]

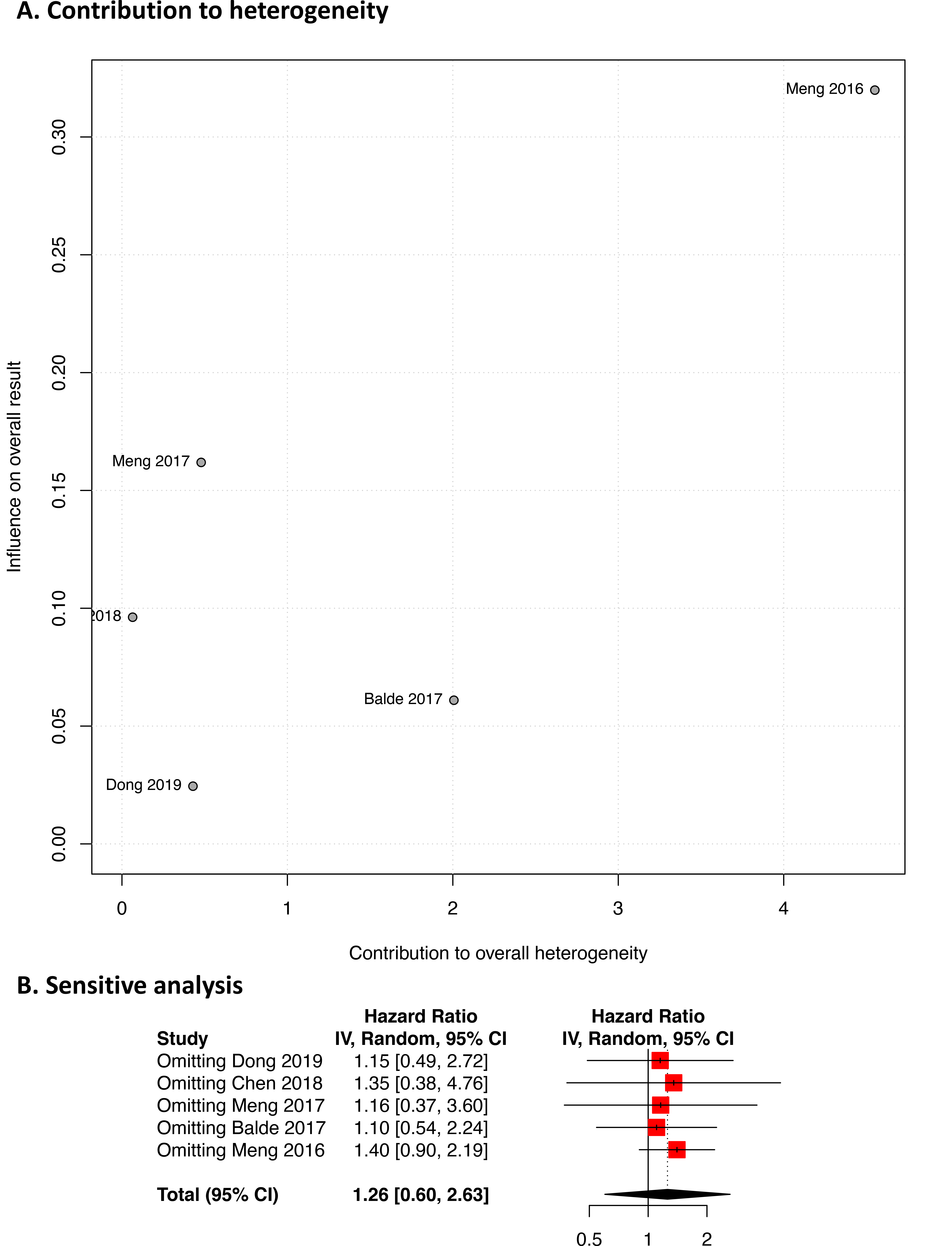

Supplement: Supplementary Figure 3 — Sensitivity analysis. [file Image_3.tif]
